# FBXW7/hCDC4 controls glioma cell proliferation *in vitro *and is a prognostic marker for survival in glioblastoma patients

**DOI:** 10.1186/1747-1028-2-9

**Published:** 2007-02-27

**Authors:** Martin Hagedorn, Maylis Delugin, Isabelle Abraldes, Nathalie Allain, Marc-Antoine Belaud-Rotureau, Michelle Turmo, Claude Prigent, Hugues Loiseau, Andréas Bikfalvi, Sophie Javerzat

**Affiliations:** 1 INSERM, E0113, Mécanismes Moléculaires de l'Angiogenèse, Talence, F-33405, France; 2 Univ Bordeaux1, Talence, F-33405, France; 3 EA2406, Histologie et Pathologie Moléculaire des Tumeurs, Univ Bordeaux 2, Bordeaux, F-33076, France; 4 CNRS, UMR6061, Génétique et Développement, Univ Rennes, Rennes, F-35043, France; 5 CHRU Bordeaux, Hôpital Pellegrin, Service de neurochirurgie, Bordeaux, F-33076, France

## Abstract

**Background:**

In the quest for novel molecular mediators of glioma progression, we studied the regulation of *FBXW*7 (h*CDC*4/h*AGO*/*SEL*10), its association with survival of patients with glioblastoma and its potential role as a tumor suppressor gene in glioma cells. The F-box protein Fbxw7 is a component of SCF^Fbxw7^, a Skp1-Cul1-F-box E3 ubiquitin ligase complex that tags specific proteins for proteasome degradation. *FBXW*7 is mutated in several human cancers and functions as a haploinsufficient tumor suppressor in mice. Any of the identified targets, Cyclin E, c-Myc, c-Jun, Notch1/4 and Aurora-A may have oncogenic properties when accumulated in tumors with *FBXW*7 loss.

**Results:**

We tested the expression of *FBXW*7 in human glioma biopsies by quantitative PCR and compared the transcript levels of grade IV glioma (glioblastoma, G-IV) with those of grade II tumors (G-II). In more than 80% G-IV, expression of *FBXW*7 was significantly reduced. In addition, levels of *FBXW*7 were correlated with survival indicating a possible implication in tumor aggressiveness. Locus 4q31.3 which carries *FBXW*7 was investigated by *in situ *hybridization on biopsy touchprints. This excluded allelic loss as the principal cause for low expression of *FBXW*7 in G-IV tumors. Two targets of Fbxw7, Aurora-A and Notch4 were preferentially immunodetected in G-IV biopsies. Next, we investigated the effects of *FBXW*7 misregulation in glioma cells. U87 cells overexpressing nuclear isoforms of Fbxw7 lose the expression of the proliferation markers PCNA and Ki-67, and get counterselected *in vitro*. This observation fits well with the hypothesis that Fbxw7 functions as a tumor suppressor in astroglial cells. Finally, *FBXW*7 knockdown in U87 cells leads to defects in mitosis that may promote aneuploidy in progressing glioma.

**Conclusion:**

Our results show that *FBXW*7 expression is a prognostic marker for patients with glioblastoma. We suggest that loss of *FBXW*7 plays an important role in glioma malignancy by allowing the accumulation of multiple oncoproteins and that interfering with Fbxw7 or its downstream targets would constitute a new therapeutic advance.

## Background

Glioblastoma (glioma grade IV, G-IV), the most common tumor arising in the central nervous system, is one of the deadliest cancers with a mean survival of less than one year [1]. Reliable molecular predictors of survival outcome as well as novel targets for efficient therapy are urgently needed to improve life conditions of patients with glioma.

In this study, we investigated the expression of *FBXW*7 (also known as h*CDC*4, h*AGO*, *SEL*10) in glioma. *FBXW*7 encodes one of the 75 F-box proteins identified so far in mammals [2]. F-box proteins represent the variable receptor component of Skp1-Cul1-F-box (SCF) complexes, that mediates binding and ubiquitination of specific proteins, which are consequently recognized and destroyed by the proteasome. In contrast to other SCF complexes such as SCF^Fbxl1 ^which target both positive and negative regulators of the cell cycle [3], all known targets of SCF^Fbxw7 ^– namely Cyclin E [4-6], c-Myc [7], c-Jun [8], Notch 1 and 4 [9-11] and Aurora-A [12] – are cell growth promoters and potential oncoproteins. Their turn-over can thus be seen as an ultimate process in tumor suppression control. Indeed, *FBXW*7 itself behaves as a haploinsufficient tumor suppressor gene: the loss of one functional allele is enough to promote epithelial tumor growth in a mouse model [13]. Given the number of its targets and the fact that *FBXW*7 is translated into three different isoforms with distinct subcellular localization, possible mechanisms of tumor suppression are bound to be complex and variable depending upon the cell type in which downregulation occurs.

*FBXW*7 is mutated in many cancer cell lines and human tumors such as endometrial, pancreatic and colorectal cancers [6,14-17]. Mutations of *FBXW*7 in brain tumors have not been investigated yet, however the corresponding locus – 4q31.3 – belongs to the most frequently lost portion of chromosome 4 in glioblastoma [18,19]. As far as targets are concerned, much attention has been focused on the misregulation of Cyclin E [20] and c-Myc [21] whereas other substrates such as Aurora-A and Notch receptors have not yet been comprehensively investigated. In tumors, correlations have been demonstrated between loss of function of *FBXW*7 and high levels of Cyclin E [20], and between Cyclin E overexpression and chromosomal instability [17]. However, the overall effect of *FBXW*7 loss in cancer cells is not straightforward and seems to vary according to cancer types [16,22]. Fujii and coworkers recently reported the accumulation of multiple targets in several *FBXW*7 mutant cell lines with a variable extent of increase in expression and even identified a different pattern of accumulation for two ovarian cancer lines, one accumulating predominantly both Cyclin E and c-Myc, the other Aurora-A solely [23].

In this study, we investigated the possible misregulation of Aurora-A and Notch 4 expression in glioma. Aurora-A is a mitotic kinase required for G2-M transition, centrosome maturation and alignment of chromosomes at metaphase. Its degradation by the proteasome occurs promptly after metaphase-anaphase transition and is necessary for mitotic exit [24]. In normal cells, the expression of Aurora-A is thus rhythmic, discrete and detected in dividing cells only. By contrast, in cancer cells, Aurora-A is frequently overexpressed indicating its possible involvement in tumorigenesis [25]. Indeed, Aurora-A overexpression results in centrosome amplification and cytokinesis failure – both promoting tetraploidization – and transformation of cells with already acquired checkpoint defects [26,27]. How does Aurora-A accumulate in cancer cells? In bladder cancer, *AURKA*/*STK*15, the gene encoding Aurora-A at locus 20q13.2 is commonly amplified and the resulting overexpression is correlated with critical clinical parameters such as invasion, metastasis and poor survival [28]. But the overexpression of Aurora-A is also frequently seen in tumors with no gain of *AURKA *at the DNA level suggesting that other deregulated mechanisms such as transcriptional activation or failure of a proteolysis component are responsible for Aurora-A accumulation. This may well apply to glioma: Klein and coworkers detected amplification of AURKA in 26% of malignant glioma while as much as 67% of the samples eventually overexpressed the gene [29].

Unlike all other targets of Fbxw7, Notch4 has not yet been investigated in human cancers though the protein is oncogenic when induced in the MMTV (mouse mammary tumor virus) mouse model [30]. Thus far, Notch4 has been described as a vascular endothelium specific signaling receptor. Together with its ligand Delta-like 4 (Dll4), it is involved in vessel development by mediating arterial/venous specification and vascular remodeling during embryogenesis [31]. In tumors, Notch4 signaling is activated in the endothelium and is here again responsible for vascular maturation [32]. On the other hand, it has been recently shown that *NOTCH*4 transcription can be derepressed in non-endothelial cells: in HeLa cells treated with endothelial growth factors, cell-type-specific AP-1 (activator protein 1) complexes are activated that are able to reprogram *NOTCH*4 expression [33]. This raises the possibility that tumor cells, which produce high amounts of proangiogenic factors – like malignant glioma cells – may ectopically express *NOTCH*4.

Here we report that expression of *FBXW*7 is strongly reduced in G-IV tumors, mostly independent of locus depletion, and that the levels of *FBXW*7 expression correlate with patient survival. We find that Aurora-A and Notch4 accumulate in perivascular zones of patient glioblastoma. Proliferation is significantly impaired in glioma cells overexpressing nuclear *FBXW*7 *in vitro *suggesting that it acts as a tumor suppressor in astroglial cells. Finally we show that knocking down *FBXW*7 in cultured glioma cells destabilizes chromosome segregation at mitosis, a process controlled by several targets of SCF^Fbxw7 ^including Aurora-A.

## Results and Discussion

### Expression of *FBXW7 *in glioma patients and correlation with survival

**Figure 1:**
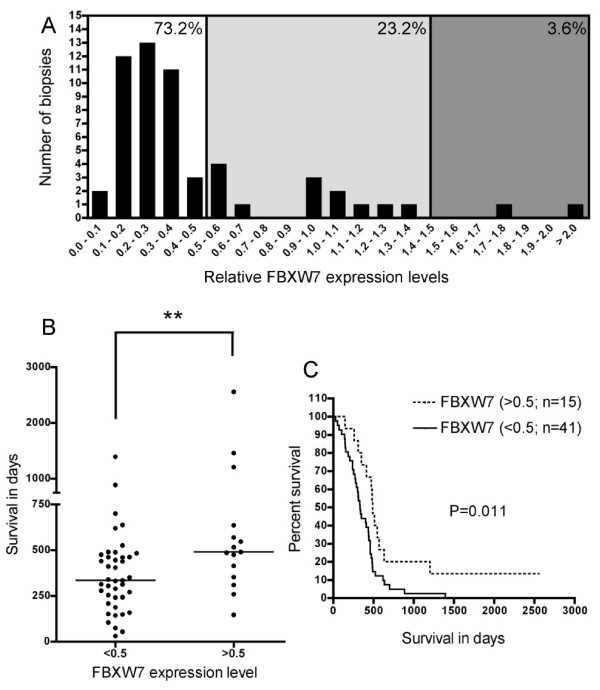
**Expression of *FBXW*7 in glioma and correlation to survival**. (A) *FBXW*7 expression was measured by quantitative RT-PCR in 56 G-IV compared to a pool of 5 G-II. 73% of high grade tumors show very significant low levels of *FBXW*7 transcripts (<0.5). (B) Relative levels of *FBXW*7 were heterogeneous, but expression defines two groups of patients with different survival times after diagnosis. The median survival in the group with *FBXW*7 levels >0.5 was 490 days, whereas patients with low expression (<0.5) had a median survival of 335 days. The difference between the two groups is significant (P = 0.0099, Mann-Whitney U-test, two-tailed). (C) Patients classified in the two groups as shown in B were subjected to Kaplan-Meier survival analysis. Survival curves for the two patient groups differed significantly (P = 0.011; Log-rank test). Note that two patients in the >0.5 group were still alive at the time the analysis were performed, more than 2000 days after initial diagnosis.

*FBXW*7 expression was evaluated by quantitative RT-PCR in 56 G-IV and 5 G-II gliomas. G-IV tumors were classified according to the normalized expression levels of *FBXW*7 compared to the mean of expression levels in G-II tumors. *FBXW*7 is significantly downregulated (<0.75) in more than 80% G-IV tumors. As illustrated in Fig. 1A, it is severely decreased of more than 2-fold (<0.5) in most samples (73,2%). Correlation was next established between survival time after initial diagnosis of glioblastoma and *FBXW*7 expression levels. As shown in Fig. 1B, expression levels were heterogeneous, but when classified into two groups (<0.5 and ≥0.5), a significant difference of survival time was highlighted (Mann-Whitney U-test, P = 0.0099). Interestingly, out of 4 tumors collected from long survivors with G-IV (> 1000 days), 3 displayed normal *FBXW*7 levels. Patients classified in the two groups as in Fig. 1B were next subjected to Kaplan-Meier survival analysis illustrating that the outcome for patients with >0.5 levels of *FBXW*7 is significantly better than for patients with reduced expression (Fig. 1C). The same study performed with two related F-box protein encoding genes, *FBXO*6 and *FBXL*1/*SKP*2, showed a similar heterogeneous spectrum of expression but no evidence for significant correlation with survival (not shown). While this study was being conducted, Bredel *et al. *described an informative gene profiling of gliomagenesis, from which *FBXW*7 emerges as a downregulated inhibitor of the MYC interacting pathway [34], reinforcing our findings that it is a highly significant predictor of aggressiveness. Our results further demonstrate that *FBXW*7 can be used as a specific prognostic marker of survival for patients with glioblastoma. Together with other recently identified molecular predictors, *FBXW*7 may enhance comprehensive classification and molecular characterization of gliomas with a likely impact on clinical management including prediction of therapeutic response.

### Allelic loss is not the primary cause for low expression of *FBXW7 *in G-IV tumors

**Figure 2:**
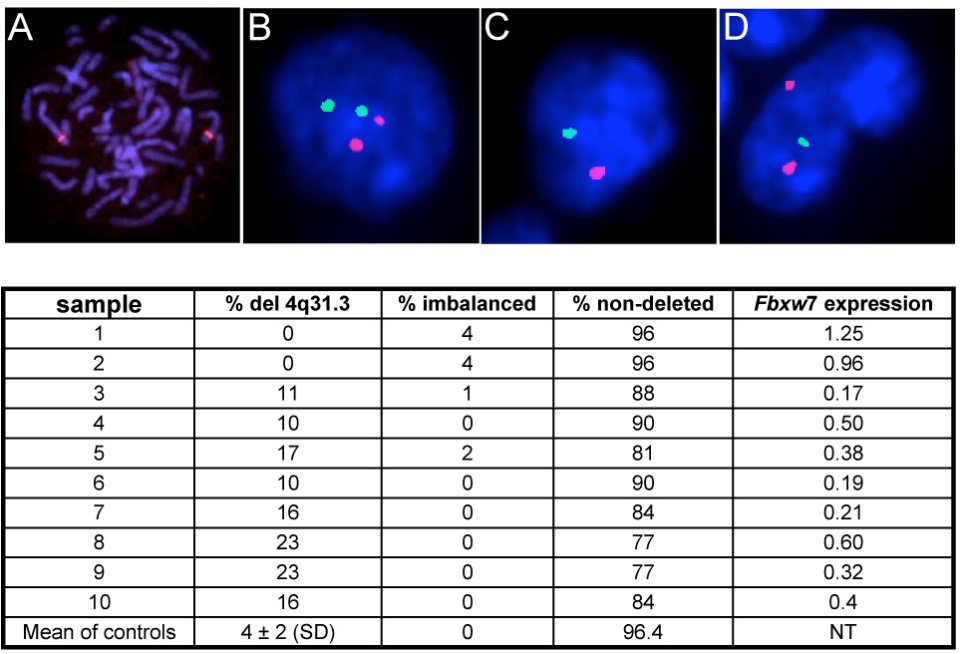
**FISH using a *FBXW*7 specific probe to detect allelic loss**. (A) Specificity of the RP11-300I24 probe containing *FBXW*7 on metaphase chromosomes from normal lymphocytes. The probe is labeled with SpectrumRed. (B-D) Dual color FISH patterns of nuclei from frozen touch prints of human glioblastoma. The 4q31.3 probe is labeled green and the CEN4 probe is labeled red. (B) normal signal, (C) monosomic for chromosome 4, (D) monoallelic deletion of 4q31.3. The table summarizes the results drawn from the FISH analysis for tumors with FBXW7 downregulation and 4q31.3 deletion (samples 3–10). Samples 1 and 2 are from tumors with no significant downregulation of *FBXW*7 expression. Control tissues were from reactive lymphadenitis. Using 10% as a cut-off value, 18/26 tumors with significant downregulation of *FBXW*7 are classified as not deleted (not detailed in the table).

In p53+/- mice, the loss of one allele of *FBXW*7 get preferentially selected in radiation-induced lymphoma cells and promotes tumor development so that *FBXW*7 meets the criteria of a haploinsufficient tumor suppressor gene [13]. In the same study, the authors show that *FBXW*7 is a direct transcriptional target of p53 itself. This raises the possibility that monoallelic deletion on its own may account for reduced expression of *FBXW*7 in patient tumors with active p53 (around 30% of primary glioblastoma [35]). *FBXW*7 maps on 4q31.3. Analyses of this region have been recently conducted in esophageal adenocarcinoma and identified deletions in 40% of tumors. The sequence of the remaining allele was wild-type in most of the cases [36]. Further studies should confirm the functional implication of monoallelic deletion (rather than loss of heterozygosity) – i.e. the haploinsufficiency of *FBXW*7 – in this type of cancer. We sought for 4q31.3 deletions in our cohort of G-IV patients. The locus specific probe containing the complete *FBXW*7 gene sequence was selected from the RP11 BAC library (clone 300I24) and proved to hybridize specifically at 4q31 on metaphase normal chromosomes (Fig. 2A). Twenty-eight tumors were subjected to touchprint dual color FISH analysis using the *FBXW*7 locus specific probe and a chromosome 4 subcentromeric probe for chromosome copy number assessment. The patterns of signal distribution in individual nuclei were collected for 50–100 cells per tumor. Representative images are shown in Fig. 2B–D. Results of this analysis are illustrated in the table of Fig. 2. Samples 1 and 2 were tumors with no significant downregulation of *FBXW*7 and did show no deletion of 4q31.3. FISH analysis identified *FBXW*7 hemizygous deletions in 31% (8/26) cases with downregulation. In these cases however, only a minority (10–23%) of cells exhibited a 4q31.3 loss while other nuclei were mainly disomic for chromosome 4 with 2 copies of 4q31.3 (see individual patterns in table of Fig. 2). FISH analysis did not detect any imbalanced case so that 69% of the tumors (18/26) were classified as non-deleted.

This analysis suggests that only for a minority of glioblastoma, monoallelic deletion of 4q31.3 may participate in reduction of *FBXW*7 expression. If downregulation is required for glioblastoma progression, these events may be selected preferentially in tumor cells that retain P53 expression. Nonetheless, we conclude that allelic loss is not a recurring cause for reduced *FBXW*7 expression in glioblastoma.

### Aurora-A and Notch4 accumulate in G-IV tumors

**Figure 3:**
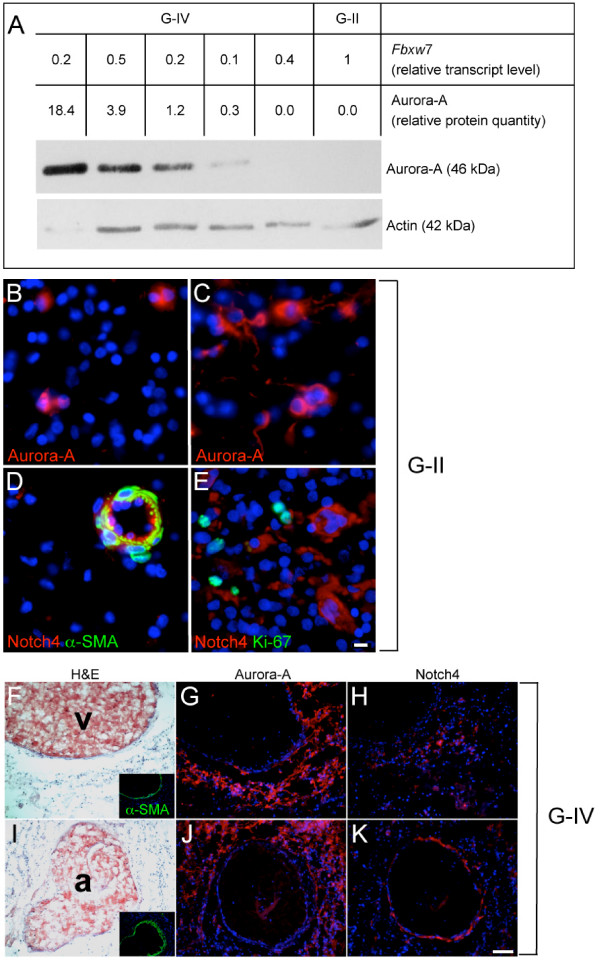
**Immunodetection of Aurora-A and Notch4, two targets of *FBXW*7**. (A) Detection of Aurora-A in human tumors by western blotting. The abundance of Aurora-A has been quantified in 5 randomly chosen G-IV and one representative G-II. The same blot has been hybridized with anti-actin antibody for loading control and normalization. Aurora-A is detected in 4 out of 5 G-IV with downregulation of *FBXW*7. (B-K) Immunohistochemical detection of Aurora-A and Notch4 in human G-II (mid panel, bar = 10 μm) and in a primary invasive glioblastoma (lower panel, bar = 100 μm). Nuclei are labeled with DAPI (blue). In G-II, Aurora-A detection (red) is mostly sparse (B) and accumulates in only a few tumor areas (C). Notch4 (red) labels only α-SMA positive (green) vascular structures (D), but not Ki-67 positive (green) proliferating tumor cells (E). By contrast, in invasive primary G-IV (lower panel) of known histology and α-SMA labeling (F, I) discriminating vascular structures (v, vein ; a, arteriole), Aurora-A is massively detected in specific zones of G-IV, mostly around vessels (G-J). Notch4 is also detected in some tumor cells in addition to the endothelial lining of arterioles (H-K).

We next asked if the loss of function of Fbxw7 protein may participate in glioma malignancy by causing accumulation of specific targets. Protein extracts from the biopsies were examined by western blotting using anti-CyclinE, anti-c-Myc, anti-Aurora-A and anti-Notch4 antibodies. Generally, levels of detected proteins were variable between samples and no significant correlation with *FBXW*7 expression could be made. It was striking however, that Aurora-A was easily detected in over 81% (35/43) of the samples with significant decrease in *FBXW*7 expression, whereas it failed under detection threshold in G-II with consistent *FBXW*7 signals (Fig. 3A). The same tendency was observed for Notch4 but neither for Cyclin E, nor c-Myc (not shown). This prompted us to investigate the presence of Aurora-A and Notch4 on sections of gliomas of different histological grades. In G-II, Aurora-A was detected sparsely in less than 5% of the tumor cells (Fig. 3B) as expected from the low proliferation index (Fig. 3E) and accumulated only in a few tumor areas (Fig. 3C). Notch4 was strongly associated with α-SMA (alpha smooth muscle cell actin) positive vessels (Fig. 3D), as expected from its previously reported role in vessel maturation [37], but was not detected in any proliferating tumor cells (Fig. 3E). In contrast, both Aurora-A and Notch4 were strongly detected in distinct areas of aggressive primary glioblastoma (Fig. 3, lower panel). Aurora-A massively accumulates in tumor cells surrounding vessels (Fig. 3G &3J). Anti-Notch4 antibody also stains tumor cells preferentially around vessels in glioblastoma in addition to the endothelial lining of α-SMA positive arterioles (Fig. 3H &3K). No accumulation of any of the two proteins was seen in poorly vascularized tumor areas (not shown).

All in all, two oncoproteins the mitotic kinase Aurora-A and the signaling receptor Notch4, which levels depend on *FBXW*7, are strongly expressed in specific zones of G-IV tumors, particularly around blood vessels.

### Overexpression of *FBXW7 *inhibits proliferation of U87 cells in vitro

**Figure 4:**
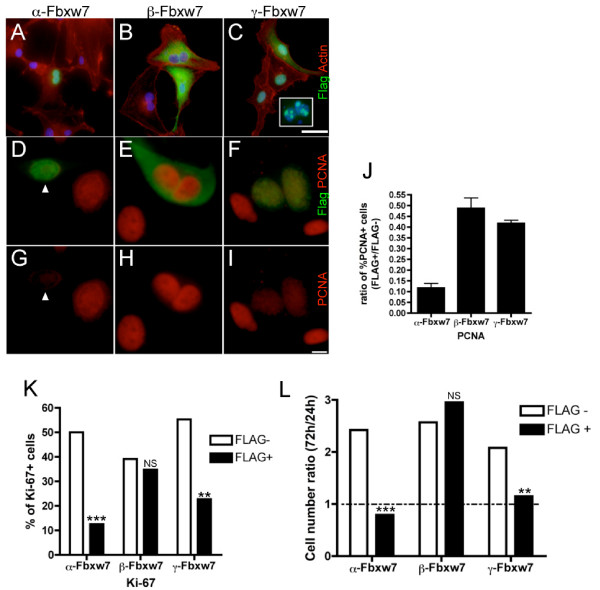
**Effect of *FBXW*7 overexpression in glioma cells**. Immunodetection of FLAG-Fbxw7 (green) in U87, 48 hours after transfection with plasmids encoding each of the three isoforms α (A), β (B), γ (C). Nuclei are labeled with DAPI (blue) and actin cytoskeleton with rhodamin-coupled phalloidin (red), bar = 50 μm. α-Fbxw7 localizes to the nucleus, β-Fbxw7 is cytoplasmic, γ-Fbxw7 localization varies. It is restricted to the nucleolus at low levels (insert) and leaks in the nucleus and in the cytoplasm at higher doses. Example of PCNA detection (red) in U87 cells overexpressing FLAG-Fbxw7 (green) α (D), β (E), γ (F). In lower panels (G-I), only the red signal in the same fields is shown. An example of α-Fbxw7-positive PCNA-negative cell is shown with arrowhead. bar = 10 μm. Quantification analysis of PCNA expression 48 h after transfection (J). Scoring was established on >150 cells per assay. The results (mean of two independent experiments, ± SEM) are expressed as the ratio of PCNA^+ ^cells in FLAG^+ ^versus FLAG^- ^cells from the same transfection well. Quantification analysis of Ki-67 expressing cells in FLAG^- ^and FLAG^+ ^cells 48 h after transfection (K). Statistical analysis was performed using the Fisher's exact test (n > 150 cells for each group). The experiment was repeated twice with similar results. Cell growth after transfection with each isoform expression plasmid (L). For each assay, cells were stained with DAPI and anti-FLAG antibody 24 h and 72 h after transfection. FLAG positive and negative cells were scored from 20 independent 40×-magnified fields and their 72 H/24 h ratio compared. Cells overexpressing nuclear isoforms α and γ are significantly counterselected. Statistical analysis was performed using the Fisher's exact test (n > 1000 cells for each assay). The experiment was repeated twice and analyzed once after 48 h with similar results.

The three isoforms of Fbxw7, α, β, and γ, have a conserved structure and molecular function but differ in their subcellular localization which is determined by the first alternative exon. Welcker and coworkers reported that, in U2OS cells, α-Fbxw7 is strongly detected in the nucleus, β-Fbxw7 in the cytoplasm and γ-Fbxw7 is mostly associated with nucleoli [21]. Hence, the three isoforms are likely to mediate degradation of specific targets in a localization-dependent manner. In order to study the effect of *FBXW*7 misregulation in glioma, U87 cells were transiently transfected with plasmids encoding each FLAG-tagged Fbxw7 isoform. Transfected cells were detected by immunofluorescence using anti-FLAG antibody. As shown in Fig. 4A–C, the subcellular localization of α-Fbxw7 and β-Fbxw7 was similar to that previously reported for other cell lines (ie, nucleoplasmic and cytoplasmic respectively) whereas isoform γ localization varied according to its level of expression: weak expression was restricted to the nucleoli whereas stronger expression was found in the cytoplasm and/or in the nucleus. We next asked whether Fbxw7 had an effect on glioma cell proliferation. Cells that express the proliferation markers PCNA or Ki-67 were scored among FLAG-positive cells compared to FLAG-negative cells. As illustrated in Fig. 4D–J, immunodetection of PCNA was specifically reduced in cells overexpressing nuclear Fbxw7. Cells overexpressing nuclear isoforms α and γ also display strongly reduced expression of Ki-67 (P < 0.0005 and P < 0.01 respectively) whereas expression of Ki-67 did not significantly change in cells overexpressing the cytoplasmic isoform β (Fig. 4K).

The effect of Fbxw7 on proliferation was further confirmed by showing that nuclear Fbxw7 counterselects transiently transfected cells in a time-course culture (Fig. 4L): the growth of cells overexpressing α-Fbxw7 or γ-Fbxw7 is strongly inhibited between 24 and 72 h after transfection (P < 0.0001 and P < 0.005 respectively) whereas FLAG negative internal control cells expand of more than 2-fold. By contrast, overexpression of isoform β does not significantly affect the growth of U87 cells. FLAG positive cells were screened for Aurora-A expression by double immunofluorescence with anti-FLAG and anti-Aurora-A antibodies. Amongst cells expressing *FBXW*7 in the nucleus (n > 400) no Aurora-A staining was detected compared to untransfected cells out of which 10% were Aurora-A positive. This mutually exclusive expression pattern suggests that Fbxw7 targets Aurora-A in glioma cells. This may interfere with cell cycle checkpoint hence the dramatic reduction of dividing cells. Given the effect of Aurora-A knockdown, we can anticipate that cells may then be arrested in the next cycle by the post-mitotic checkpoint [25], hence their progressive counterselection in culture.

In summary, these results indicate that nuclear Fbxw7 exerts an inhibitory effect on proliferation of glioma cells *in vitro *and therefore support the hypothesis that loss of nuclear *FBXW*7 expression might contribute to tumor progression in patients with astroglial tumors.

### Fbxw7 knockdown causes mitotic defects in U87 cells

**Figure 5:**
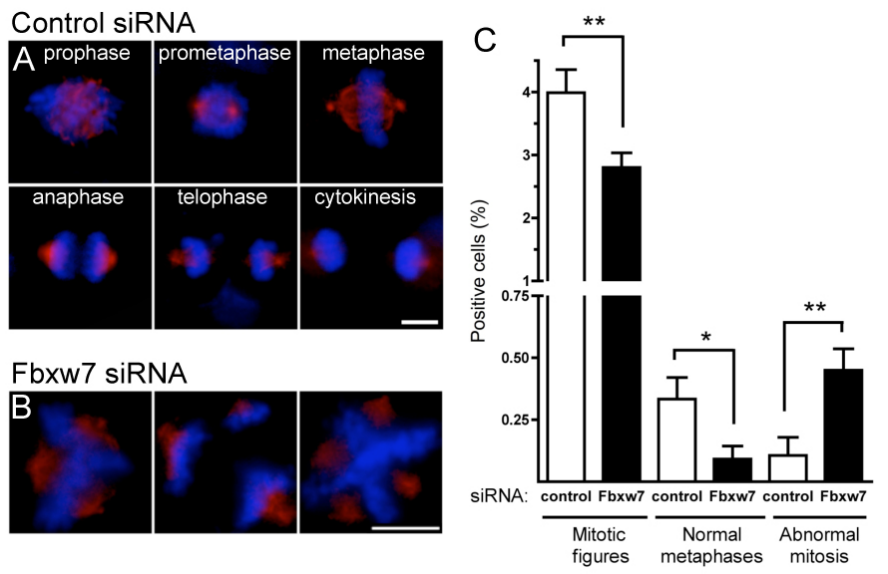
**Effect of *FBXW*7 knockdown in glioma cells**. Normal mitosis figures in control siRNA transfected cells (A) and examples of abnormal mitosis in U87 cells transfected with siRNA against *FBXW*7 (B). Chromosomes are stained with DAPI (blue) and spindle poles with anti-Aurora-A antibody (red), bar = 10 μm. Quantification of abnormal mitosis detected as in B, 48 h after transfection with siRNA against *FBXW*7. More than 3400 cells were analyzed for each condition. Aurora-A positive cells as in A and B were classified as mitotic and accounted for around 4% of total cells in controls. *FBXW*7 siRNA-transfected cells showed significantly less mitotic figures (72.2% of controls, P = 0.0094), fewer normal metaphases (27.5% of controls, P = 0.022), but much more abnormal mitoses (7.3 fold compared to controls, P = 0.0042; unpaired t-test, two-tailed).

In order to examine the consequences of *FBXW*7 downregulation in glioma, U87 cells – that express low levels of *FBXW*7 in culture (not shown) – were transfected with previously described specific siRNAs directed against a shared exon of *FBXW*7 [20]. Cells transfected with either control siRNA or *FBXW*7 siRNA were grown for 48 hours and stained with DAPI and anti-Aurora-A antibody. Nuclei with individualized chromosomes and Aurora-A positive staining [24] were classified as mitotic and accounted for at least 4% of cells in independent control experiments. We compared mitotic figures in *FBXW*7 knockdown cells to control cells (>100 mitosis for each group) and found that 98.5% of the figures were normal in controls and could be assigned to a specific phase as in Fig. 5A. By contrast, as much as 16% of mitosis in *FBXW*7 knockdown cells were seen with 3 or 4 spindle poles (Fig. 5B), and there was significantly less normal metaphases (Fig. 5C). Unexpectedly, the number of mitotic figures was significantly decreased in *FBXW*7 knockdown cells. This suggests that in glioma cells, loss of Fbxw7 function primarily triggers mitotic defects that could select for aneuploid cells with growth advantage in the evolving tumor.

Possible targets that accumulation may cause checkpoint disturbances and chromosomal instability are Aurora-A and Cyclin E although they could not be quantitatively monitored in these transient transfection assays. Cyclin E accumulation has been shown to raise similar defects in colorectal cancer cells [17] and is accumulated in tumors of different types [20] including glioma with poor prognosis [38].

## Conclusion

In this study, we have shown that the expression of *FBXW*7 is strongly reduced in glioblastoma. Moreover, *FBXW*7 emerges as a relevant survival marker for patients with glioblastoma that warrants further validation in the clinic. Our results suggest that *FBXW*7 downregulation promotes gliomagenesis via the accumulation of oncogenic cell cycle regulators that control cell division such as Aurora-A and/or Cyclin E or Notch. The contribution of each of the targets remains to be investigated in details in glioma.

Gene therapy with *FBXW*7 is already foreseen for cancer [23]. Glioblastoma patients should benefit of an ameliorated survival prognosis based on *FBXW*7 quantification. Future studies will tell if there is also a way to improve therapy based on Fbxw7 rescue for those with the most dismal prognosis.

## Methods

### Cells and tissue collection

U87 human cells glioma (ATCC/LGCpromochem, Molsheim, France) were maintained in DMEM with 10% FBS, antibiotics, and L-glutamine. Glioma biopsies were classified according to the World Health Organization [39], the median survival of the G-IV group was 379 days (n = 54 patients, excluding 2 patients still alive at the time of analysis). Samples were immediately snap-frozen and stored until further use for protein and RNA purification, tissue sectioning, and touch printing of interphase nuclei. All procedures complied with current French laws.

### Fluorescence *in situ *hybridization (FISH) on interphase tumor nuclei

The *FBXW*7 probe contained in BAC clone RP-11 300I24 was labeled by nick translation with SpectrumGreen dUTP (Abbott-Vysis, France) and co-hybridized with a chromosome 4 alpha-satellite probe (D4Z1) coupled to rhodamin (Qbiogene, Illkirch, France). Efficiency and specificity were assessed on metaphases and interphases from cultured human lymphocytes. Dual color FISH analysis were performed on touchprints of frozen tissues as previously described [40]. The slides were then fixed by immersion in methanol and methanol/acetic acid baths. After drying and dehydratation, the probes were added to each slide. A coverslip was placed over each hybridization slide and sealed with rubber cement. Slides and probes were co-denatured using the Hybrite Hybridization System (Abbott-Vysis, Rungis, France) at 73°C during 5 min. Hybridization was performed 24 h at 37°C in a humidified box. Finally, slides were washed, dehydrated and nuclei were counterstained with 4,6-diamidino-2-phenyindole (DAPI) diluted in Vectashield (Abcys, Paris, France). The microscopic analysis was done by two independent observers using a fluorescent Axioplan II microscope (Zeiss, Le Pecq, France). A minimum of 50 tumour cell nuclei were evaluated for each slide. Hybridization signals of control (centromere of chromosome 4, CEN4) and test (*FBXW*7) probes were counted for each nucleus. Nuclei were then classified either as 1) deleted (ratio of control and test probes 2/1, 4/2, 3/1, 4/1,...), 2) imbalanced (disproportion of the ratio of control and test probes such as 3/2, 4/3, 5/3, etc...), or 3) non-deleted (equal ratio of control and test probes signals). The cut-off value was determined as the mean + three standard deviations of the percentage of deleted nuclei on control tissues [41]. Finally, a tumor was classified either as deleted (% of deleted nuclei ≥ cut-off), or as non-deleted (% of deleted + imbalanced nuclei < cut-off) or as imbalanced (% of imbalanced nuclei or sum of imbalanced + deleted nuclei ≥ cut-off).

### Real-time quantitative PCR (qPCR)

RNA was purified from biopsies or cultured cells using RNeasy columns (Qiagen, Courtaboeuf Cedex, France). RNA quality was checked by electrophoresis and any sample displaying degraded RNA was excluded from the study. RNA was reversed-transcribed with SuperScript II RNase H-Reverse Transcriptase (Invitrogen, Cergy Pontoise Cedex, France) by using oligo (dT)_15 _priming. Human-specific primers for qPCR were designed and evaluated for amplification efficiency. Primer sequences were: α-tubulin (NM_006082), 5'-GAGTGCATCTCCATCCACGTT-3', 5'-TAGAGCTCCCAGCAGGCATT-3', *FBXW*7 (target sequence common to all isoforms), 5'-CCACTGGGCTTGTACCATGTT-3', 5'-CAGATGTAATTCGGCGTCGTT-3'. Real-time PCR was carried out in a MX3000P thermocycler (Stratagene) by using SYBR Green dye (ABgene, Courtaboeuf Cedex; France). FBXW7 expression was steady in all G-II samples tested (mean ΔCt = 6.27, SD = 1). Hence, five controls were individually run in parallel with every G-IV PCR assay, and the mean ΔCt served as normalization value. Normalization and quantification were calculated as described previously [42]. All G-IV samples were tested in a minimum of two independent experiments.

### Cell transfection

U87 cells were seeded in LabTek chambers (100000 cells per well) and transfected the following day with 100 ng (α) or 500 ng (β and γ) of plasmid containing FLAG-*FBXW*7 cDNA [21], Lipofectamine and Reagent Plus (Invitrogen). For knockdown experiments, human *FBXW*7 specific [20] and negative control siRNAs were purchased from Eurogentec (Angers, France) and used at a concentration of 100 nM as previously described [43]. Knockdown was verified 48 h after transfection by real-time PCR as described above.

### Western blotting and Immunohistochemistry

Primary antibodies were mouse monoclonal anti-FLAG (Sigma, clone M2), anti-Aurora-A [44], anti-α-SMA (DakoCytomation, clone M0851), anti-Ki-67 (DakoCytomation, clone M7240), anti-PCNA (Santa Cruz sc-56), rabbit polyclonal anti-FLAG (Sigma), anti-Notch4 (Santa Cruz, clone H-225), goat polyclonal anti-actin (Santa Cruz, clone I-19),

For western blotting, 20 μg of proteins from each tumor were separated on a 15% SDS-polyacrylamide gel, then electrotransferred onto a Hybond-S membrane (Amersham, Les Ulis, France). Membranes were blocked in PBST containing 5% skim milk for 2 h at 4°C, and incubated with primary antibodies at 1:200 dilution. After washing, immunocomplexes were identified with secondary antibodies coupled to peroxidase. The blots were visualized using chemiluminescence (ECL, Amersham – GE Healthcare Europe, Orsay, France). Immunofluorescence was carried out on 10 μm cryosections of tumors or on U87 cells grown in Labtek chambers. Tissue samples and cells were fixed in paraformaldehyde, formaline-ethanol (PCNA detection) or methanol-acetone (Aurora-A) and incubated after saturation with the primary antibodies at the concentration recommended by the supplier. After washing, slides were incubated with secondary antibodies (Alexa Fluor 488, and 546; 1:2000, Molecular Probes – Invitrogen) and mounted with Vectashield containing DAPI for nuclei staining.

### Statistical analysis

All statistical analysis and graphs were performed using GraphPad Prism software.

## Competing interests

The author(s) declare that they have no competing interests.

## Authors' contributions

MD performed qPCR and western blotting analysis. IA and NA performed cell transfections and immunofluorescence experiments. HL provided biopsies and all related detailed informations. CP provided the Aurora-A monoclonal antibody. MT and MABR performed and analyzed the FISH on tumor touchprints. MH performed statistical analysis. MH and SJ are the principal investigators who designed, supervised and analyzed the study; SJ edited the manuscript. All authors read and approved the final manuscript.

## References

[B1] Maher EA, Furnari FB, Bachoo RM, Rowitch DH, Louis DN, Cavenee WK, DePinho RA (2001). Malignant glioma: genetics and biology of a grave matter. Genes Dev.

[B2] Jin J, Cardozo T, Lovering RC, Elledge SJ, Pagano M, Harper JW (2004). Systematic analysis and nomenclature of mammalian F-box proteins. Genes Dev.

[B3] Nakayama KI, Nakayama K (2005). Regulation of the cell cycle by SCF-type ubiquitin ligases. Semin Cell Dev Biol.

[B4] Koepp DM, Schaefer LK, Ye X, Keyomarsi K, Chu C, Harper JW, Elledge SJ (2001). Phosphorylation-dependent ubiquitination of cyclin E by the SCFFbw7 ubiquitin ligase. Science.

[B5] Moberg KH, Bell DW, Wahrer DC, Haber DA, Hariharan IK (2001). Archipelago regulates Cyclin E levels in Drosophila and is mutated in human cancer cell lines. Nature.

[B6] Strohmaier H, Spruck CH, Kaiser P, Won KA, Sangfelt O, Reed SI (2001). Human F-box protein hCdc4 targets cyclin E for proteolysis and is mutated in a breast cancer cell line. Nature.

[B7] Yada M, Hatakeyama S, Kamura T, Nishiyama M, Tsunematsu R, Imaki H, Ishida N, Okumura F, Nakayama K, Nakayama KI (2004). Phosphorylation-dependent degradation of c-Myc is mediated by the F-box protein Fbw7. Embo J.

[B8] Nateri AS, Riera-Sans L, Da Costa C, Behrens A (2004). The ubiquitin ligase SCFFbw7 antagonizes apoptotic JNK signaling. Science.

[B9] Gupta-Rossi N, Le Bail O, Gonen H, Brou C, Logeat F, Six E, Ciechanover A, Israel A (2001). Functional interaction between SEL-10, an F-box protein, and the nuclear form of activated Notch1 receptor. J Biol Chem.

[B10] Oberg C, Li J, Pauley A, Wolf E, Gurney M, Lendahl U (2001). The Notch intracellular domain is ubiquitinated and negatively regulated by the mammalian Sel-10 homolog. J Biol Chem.

[B11] Wu G, Lyapina S, Das I, Li J, Gurney M, Pauley A, Chui I, Deshaies RJ, Kitajewski J (2001). SEL-10 is an inhibitor of notch signaling that targets notch for ubiquitin-mediated protein degradation. Mol Cell Biol.

[B12] Minella AC, Clurman BE (2005). Mechanisms of tumor suppression by the SCF(Fbw7). Cell Cycle.

[B13] Mao JH, Perez-Losada J, Wu D, Delrosario R, Tsunematsu R, Nakayama KI, Brown K, Bryson S, Balmain A (2004). Fbxw7/Cdc4 is a p53-dependent, haploinsufficient tumour suppressor gene. Nature.

[B14] Spruck CH, Strohmaier H, Sangfelt O, Muller HM, Hubalek M, Muller-Holzner E, Marth C, Widschwendter M, Reed SI (2002). hCDC4 gene mutations in endometrial cancer. Cancer Res.

[B15] Calhoun ES, Jones JB, Ashfaq R, Adsay V, Baker SJ, Valentine V, Hempen PM, Hilgers W, Yeo CJ, Hruban RH, Kern SE (2003). BRAF and FBXW7 (CDC4, FBW7, AGO, SEL10) mutations in distinct subsets of pancreatic cancer: potential therapeutic targets. Am J Pathol.

[B16] Kemp Z, Rowan A, Chambers W, Wortham N, Halford S, Sieber O, Mortensen N, von Herbay A, Gunther T, Ilyas M, Tomlinson I (2005). CDC4 mutations occur in a subset of colorectal cancers but are not predicted to cause loss of function and are not associated with chromosomal instability. Cancer Res.

[B17] Rajagopalan H, Jallepalli PV, Rago C, Velculescu VE, Kinzler KW, Vogelstein B, Lengauer C (2004). Inactivation of hCDC4 can cause chromosomal instability. Nature.

[B18] Huhn SL, Mohapatra G, Bollen A, Lamborn K, Prados MD, Feuerstein BG (1999). Chromosomal abnormalities in glioblastoma multiforme by comparative genomic hybridization: correlation with radiation treatment outcome. Clin Cancer Res.

[B19] Mulholland PJ, Fiegler H, Mazzanti C, Gorman P, Sasieni P, Adams J, Jones TA, Babbage JW, Vatcheva R, Ichimura K, East P, Poullikas C, Collins VP, Carter NP, Tomlinson IP, Sheer D (2006). Genomic profiling identifies discrete deletions associated with translocations in glioblastoma multiforme. Cell Cycle.

[B20] Ekholm-Reed S, Spruck CH, Sangfelt O, van Drogen F, Mueller-Holzner E, Widschwendter M, Zetterberg A, Reed SI (2004). Mutation of hCDC4 leads to cell cycle deregulation of cyclin E in cancer. Cancer Res.

[B21] Welcker M, Orian A, Grim JA, Eisenman RN, Clurman BE (2004). A nucleolar isoform of the Fbw7 ubiquitin ligase regulates c-Myc and cell size. Curr Biol.

[B22] Willmarth NE, Albertson DG, Ethier SP (2004). Chromosomal instability and lack of cyclin E regulation in hCdc4 mutant human breast cancer cells. Breast Cancer Res.

[B23] Fujii Y, Yada M, Nishiyama M, Kamura T, Takahashi H, Tsunematsu R, Susaki E, Nakagawa T, Matsumoto A, Nakayama KI (2006). Fbxw7 contributes to tumor suppression by targeting multiple proteins for ubiquitin-dependent degradation. Cancer Sci.

[B24] Marumoto T, Zhang D, Saya H (2005). Aurora-A – a guardian of poles. Nat Rev Cancer.

[B25] Giet R, Petretti C, Prigent C (2005). Aurora kinases, aneuploidy and cancer, a coincidence or a real link?. Trends Cell Biol.

[B26] Zhou H, Kuang J, Zhong L, Kuo WL, Gray JW, Sahin A, Brinkley BR, Sen S (1998). Tumour amplified kinase STK15/BTAK induces centrosome amplification, aneuploidy and transformation. Nat Genet.

[B27] Meraldi P, Honda R, Nigg EA (2002). Aurora-A overexpression reveals tetraploidization as a major route to centrosome amplification in p53-/-cells. Embo J.

[B28] Sen S, Zhou H, Zhang RD, Yoon DS, Vakar-Lopez F, Ito S, Jiang F, Johnston D, Grossman HB, Ruifrok AC, Katz RL, Brinkley W, Czerniak B (2002). Amplification/overexpression of a mitotic kinase gene in human bladder cancer. J Natl Cancer Inst.

[B29] Klein A, Reichardt W, Jung V, Zang KD, Meese E, Urbschat S (2004). Overexpression and amplification of STK15 in human gliomas. Int J Oncol.

[B30] Politi K, Feirt N, Kitajewski J (2004). Notch in mammary gland development and breast cancer. Semin Cancer Biol.

[B31] Shawber CJ, Kitajewski J (2004). Notch function in the vasculature: insights from zebrafish, mouse and man. Bioessays.

[B32] Hainaud P, Contreres JO, Villemain A, Liu LX, Plouet J, Tobelem G, Dupuy E (2006). The Role of the Vascular Endothelial Growth Factor-Delta-like 4 Ligand/Notch4-Ephrin B2 Cascade in Tumor Vessel Remodeling and Endothelial Cell Functions. Cancer Res.

[B33] Wu J, Iwata F, Grass JA, Osborne CS, Elnitski L, Fraser P, Ohneda O, Yamamoto M, Bresnick EH (2005). Molecular determinants of NOTCH4 transcription in vascular endothelium. Mol Cell Biol.

[B34] Bredel M, Bredel C, Juric D, Harsh GR, Vogel H, Recht LD, Sikic BI (2005). Functional network analysis reveals extended gliomagenesis pathway maps and three novel MYC-interacting genes in human gliomas. Cancer Res.

[B35] Houillier C, Lejeune J, Benouaich-Amiel A, Laigle-Donadey F, Criniere E, Mokhtari K, Thillet J, Delattre JY, Hoang-Xuan K, Sanson M (2006). Prognostic impact of molecular markers in a series of 220 primary glioblastomas. Cancer.

[B36] Sterian A, Kan T, Berki AT, Mori Y, Olaru A, Schulmann K, Sato F, Wang S, Paun B, Cai K, Hamilton JP, Abraham JM, Meltzer SJ (2006). Mutational and LOH analyses of the chromosome 4q region in esophageal adenocarcinoma. Oncology.

[B37] Villa N, Walker L, Lindsell CE, Gasson J, Iruela-Arispe ML, Weinmaster G (2001). Vascular expression of Notch pathway receptors and ligands is restricted to arterial vessels. Mech Dev.

[B38] Tamiya T, Mizumatsu S, Ono Y, Abe T, Matsumoto K, Furuta T, Ohmoto T (2001). High cyclin E/low p27Kip1 expression is associated with poor prognosis in astrocytomas. Acta Neuropathol (Berl).

[B39] Kleihues P, Louis DN, Scheithauer BW, Rorke LB, Reifenberger G, Burger PC, Cavenee WK (2002). The WHO classification of tumors of the nervous system. J Neuropathol Exp Neurol.

[B40] Belaud-Rotureau MA, Meunier N, Eimer S, Vital A, Loiseau H, Merlio JP (2006). Automatized assessment of 1p36-19q13 status in gliomas by interphase FISH assay on touch imprints of frozen tumours. Acta Neuropathol (Berl).

[B41] Remstein ED, Kurtin PJ, Buno I, Bailey RJ, Proffitt J, Wyatt WA, Hanson CA, Dewald GW (2000). Diagnostic utility of fluorescence in situ hybridization in mantle-cell lymphoma. Br J Haematol.

[B42] Hagedorn M, Javerzat S, Gilges D, Meyre A, de Lafarge B, Eichmann A, Bikfalvi A (2005). Accessing key steps of human tumor progression in vivo by using an avian embryo model. Proc Natl Acad Sci USA.

[B43] Takei Y, Kadomatsu K, Yuzawa Y, Matsuo S, Muramatsu T (2004). A small interfering RNA targeting vascular endothelial growth factor as cancer therapeutics. Cancer Res.

[B44] Cremet JY, Descamps S, Verite F, Martin A, Prigent C (2003). Preparation and characterization of a human aurora-A kinase monoclonal antibody. Mol Cell Biochem.

